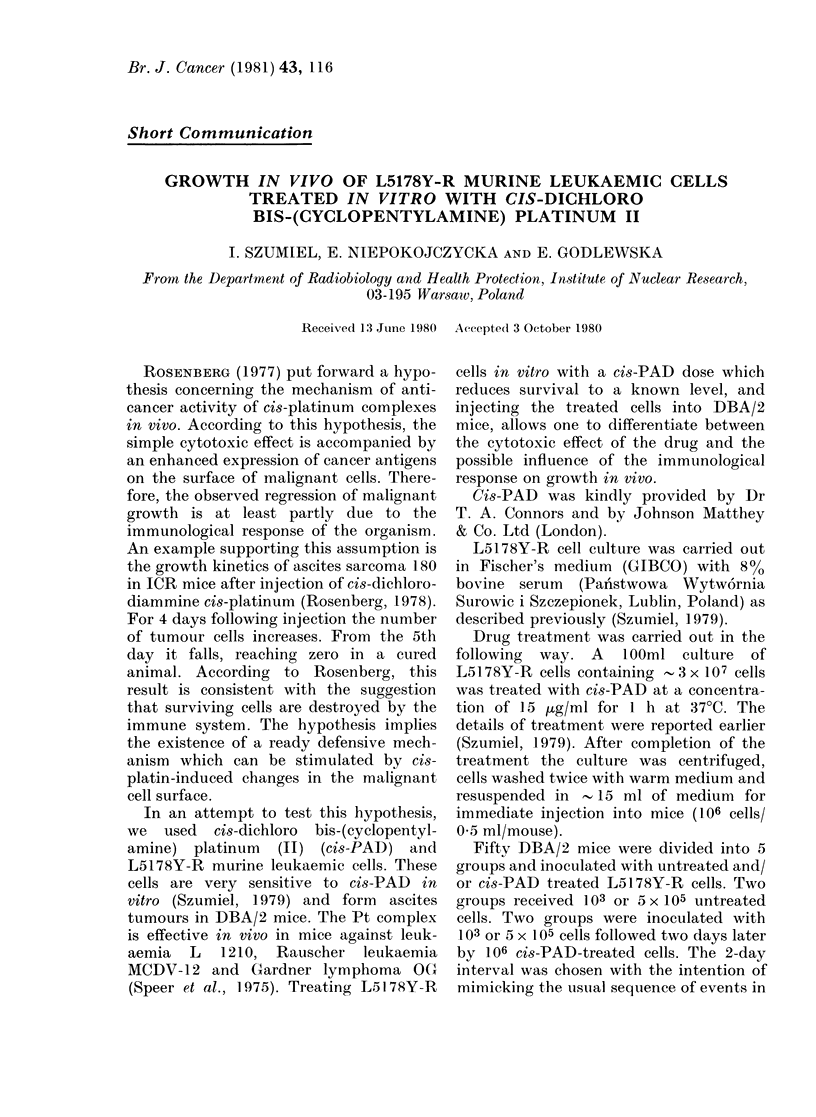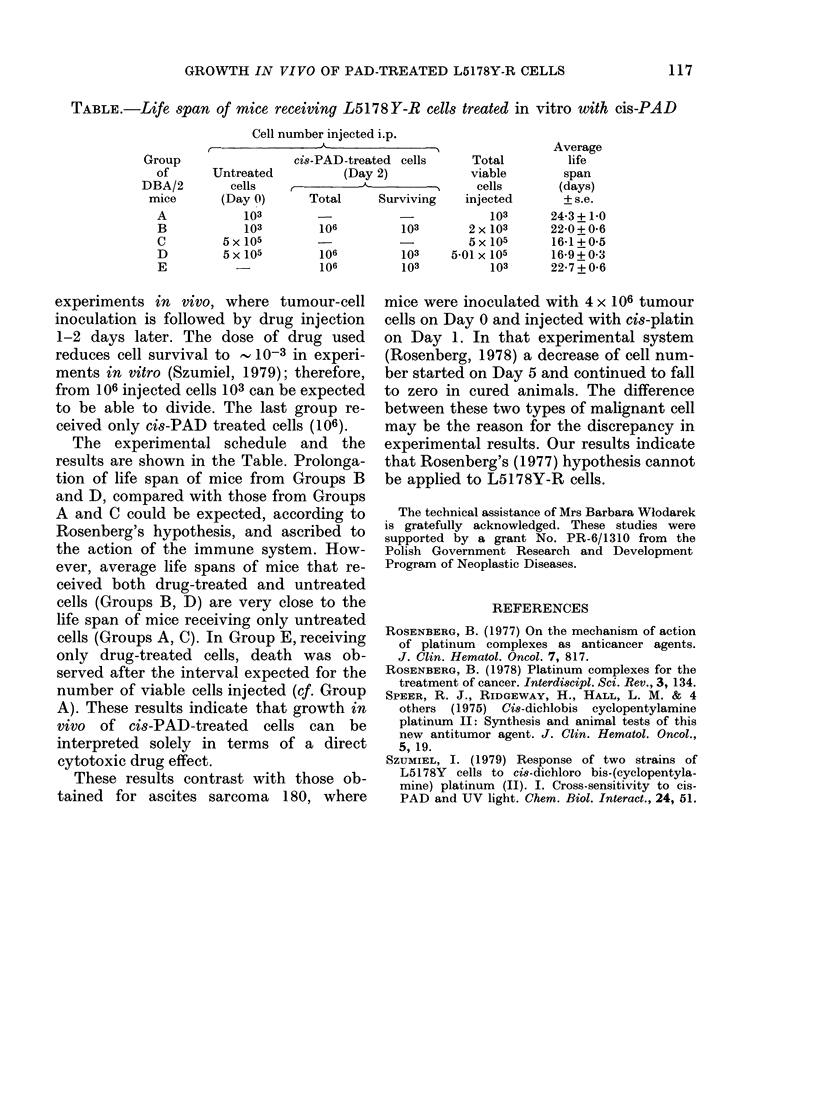# Growth in vivo of l5178y-r murine leukaemic cells treated in vitro with cis-dichloro bis-(cyclopentylamine) platinum II.

**DOI:** 10.1038/bjc.1981.18

**Published:** 1981-01

**Authors:** I. Szumiel, E. Niepokojczycka, E. Godlewska


*Br. J. Cancer* (1981) **43**, 116

***Short Communication***

### GROWTH *IN VIVO* OF L5178Y-R MURINE LEUKAEMIC CELLS TREATED *IN VITRO* WITH *CIS*-DICHLORO BIS-(CYCLOPENTYLAMINE) PLATINUM II

I. SZUMIEL, E. NIEPOKOJCZYCKA AND E. GODLEWSKA

*From the Department of Radiobiology and Health Protection, Institute of Nuclear Research, 03-195 Warsaw, Poland*

Received 13 June 1980 Accepted 3 October 1980

ROSENBERG (1977) put forward a hypothesis concerning the mechanism of anticancer activity of *cis*-platinum complexes *in vivo*. According to this hypothesis, the simple cytotoxic effect is accompanied by an enhanced expression of cancer antigens on the surface of malignant cells. Therefore, the observed regression of malignant growth is at least partly due to the immunological response of the organism. An example supporting this assumption is the growth kinetics of ascites sarcoma 180 in ICR mice after injection of *cis*-dichlorodiammine *cis*-platinum (Rosenberg, 1978). For 4 days following injection the number of tumour cells increases. From the 5th day it falls, reaching zero in a cured animal. According to Rosenberg, this result is consistent with the suggestion that surviving cells are destroyed by the immune system. The hypothesis implies the existence of a ready defensive mechanism which can be stimulated by *cis*-platin-induced changes in the malignant cell surface.

In an attempt to test this hypothesis, we used *cis*-dichloro bis-(cyclopentylamine) platinum (II) (*cis*-PAD) and L5178Y-R murine leukaemic cells. These cells are very sensitive to *cis*-PAD *in vitro* (Szumiel, 1979) and form ascites tumours in DBA/2 mice. The Pt complex is effective *in vivo* in mice against leukaemia L 1210, Rauscher leukaemia MCDV-12 and Gardner lymphoma OG (Speer *et al.*, 1975). Treating L5178Y-R cells *in vitro* with a *cis*-PAD dose which reduces survival to a known level, and injecting the treated cells into DBA/2 mice, allows one to differentiate between the cytotoxic effect of the drug and the possible influence of the immunological response on growth *in vivo*.

*Cis*-PAD was kindly provided by Dr T. A. Connors and by Johnson Matthey & Co. Ltd (London).

L5178Y-R cell culture was carried out in Fischer's medium (GIBCO) with 8% bovine serum (Państwowa Wytwórnia Surowic i Szczepionek, Lublin, Poland) as described previously (Szumiel, 1979).

Drug treatment was carried out in the following way. A 100ml culture of L5178Y-R cells containing $\sim 3 \times 10^7$ cells was treated with *cis*-PAD at a concentration of 15 $\mu$g/ml for 1 h at 37°C. The details of treatment were reported earlier (Szumiel, 1979). After completion of the treatment the culture was centrifuged, cells washed twice with warm medium and resuspended in $\sim 15$ ml of medium for immediate injection into mice ($10^6$ cells/0·5 ml/mouse).

Fifty DBA/2 mice were divided into 5 groups and inoculated with untreated and/or *cis*-PAD treated L5178Y-R cells. Two groups received $10^3$ or $5 \times 10^5$ untreated cells. Two groups were inoculated with $10^3$ or $5 \times 10^5$ cells followed two days later by $10^6$ *cis*-PAD-treated cells. The 2-day interval was chosen with the intention of mimicking the usual sequence of events in

TABLE.—*Life span of mice receiving L5178Y-R cells treated* in vitro *with* cis-*PAD*

| Group of DBA/2 mice | Cell number injected i.p. Untreated cells (Day 0) | *cis*-PAD-treated cells (Day 2) Total | Surviving | Total viable cells injected | Average life span (days) ± s.e. |
|---|---|---|---|---|---|
| A | $10^3$ | — | — | $10^3$ | 24·3 ± 1·0 |
| B | $10^3$ | $10^6$ | $10^3$ | $2 \times 10^3$ | 22·0 ± 0·6 |
| C | $5 \times 10^5$ | — | — | $5 \times 10^5$ | 16·1 ± 0·5 |
| D | $5 \times 10^5$ | $10^6$ | $10^3$ | $5{\cdot}01 \times 10^5$ | 16·9 ± 0·3 |
| E | — | $10^6$ | $10^3$ | $10^3$ | 22·7 ± 0·6 |

experiments *in vivo*, where tumour-cell inoculation is followed by drug injection 1–2 days later. The dose of drug used reduces cell survival to $\sim 10^{-3}$ in experiments *in vitro* (Szumiel, 1979); therefore, from $10^6$ injected cells $10^3$ can be expected to be able to divide. The last group received only *cis*-PAD treated cells ($10^6$).

The experimental schedule and the results are shown in the Table. Prolongation of life span of mice from Groups B and D, compared with those from Groups A and C could be expected, according to Rosenberg's hypothesis, and ascribed to the action of the immune system. However, average life spans of mice that received both drug-treated and untreated cells (Groups B, D) are very close to the life span of mice receiving only untreated cells (Groups A, C). In Group E, receiving only drug-treated cells, death was observed after the interval expected for the number of viable cells injected (*cf.* Group A). These results indicate that growth *in vivo* of *cis*-PAD-treated cells can be interpreted solely in terms of a direct cytotoxic drug effect.

These results contrast with those obtained for ascites sarcoma 180, where mice were inoculated with $4 \times 10^6$ tumour cells on Day 0 and injected with *cis*-platin on Day 1. In that experimental system (Rosenberg, 1978) a decrease of cell number started on Day 5 and continued to fall to zero in cured animals. The difference between these two types of malignant cell may be the reason for the discrepancy in experimental results. Our results indicate that Rosenberg's (1977) hypothesis cannot be applied to L5178Y-R cells.

The technical assistance of Mrs Barbara Włodarek is gratefully acknowledged. These studies were supported by a grant No. PR-6/1310 from the Polish Government Research and Development Program of Neoplastic Diseases.

### REFERENCES

ROSENBERG, B. (1977) On the mechanism of action of platinum complexes as anticancer agents. *J. Clin. Hematol. Oncol.* **7**, 817.

ROSENBERG, B. (1978) Platinum complexes for the treatment of cancer. *Interdiscipl. Sci. Rev.*, **3**, 134.

SPEER, R. J., RIDGEWAY, H., HALL, L. M. & 4 others (1975) *Cis*-dichlobis cyclopentylamine platinum II: Synthesis and animal tests of this new antitumor agent. *J. Clin. Hematol. Oncol.*, **5**, 19.

SZUMIEL, I. (1979) Response of two strains of L5178Y cells to *cis*-dichloro bis-(cyclopentylamine) platinum (II). I. Cross-sensitivity to cis-PAD and UV light. *Chem. Biol. Interact.*, **24**, 51.